# Adaptive Optics Reveals Photoreceptor Abnormalities in Diabetic Macular Ischemia

**DOI:** 10.1371/journal.pone.0169926

**Published:** 2017-01-09

**Authors:** Peter L. Nesper, Fabio Scarinci, Amani A. Fawzi

**Affiliations:** 1 Department of Ophthalmology, Feinberg School of Medicine, Northwestern University, Chicago, Illinois, United States of America; 2 G.B. Bietti Eye Foundation-IRCCS, Rome, Italy; University of Florida, UNITED STATES

## Abstract

Diabetic macular ischemia (DMI) is a phenotype of diabetic retinopathy (DR) associated with chronic hypoxia of retinal tissue. The goal of this prospective observational study was to report evidence of photoreceptor abnormalities using adaptive optics scanning laser ophthalmoscopy (AOSLO) in eyes with DR in the setting of deep capillary plexus (DCP) non-perfusion. Eleven eyes from 11 patients (6 women, age 31–68), diagnosed with DR without macular edema, underwent optical coherence tomography angiography (OCTA) and AOSLO imaging. One patient without OCTA imaging underwent fluorescein angiography to characterize the enlargement of the foveal avascular zone. The parameters studied included photoreceptor heterogeneity packing index (HPi) on AOSLO, as well as DCP non-perfusion and vessel density on OCTA. Using AOSLO, OCTA and spectral domain (SD)-OCT, we observed that photoreceptor abnormalities on AOSLO and SD-OCT were found in eyes with non-perfusion of the DCP on OCTA. All eight eyes with DCP non-flow on OCTA showed photoreceptor abnormalities on AOSLO. Six of the eight eyes also had outer retinal abnormalities on SD-OCT. Three eyes with DR and robust capillary perfusion of the DCP had normal photoreceptors on SD-OCT and AOSLO. Compared to eyes with DR without DCP non-flow, the eight eyes with DCP non-flow had significantly lower HPi (P = 0.013) and parafoveal DCP vessel density (P = 0.016). We found a significant correlation between cone HPi and parafoveal DCP vessel density (r = 0.681, *P* = 0.030). Using a novel approach with AOSLO and OCTA, this study shows an association between capillary non-perfusion of the DCP and abnormalities in the photoreceptor layer in eyes with DR. This observation is important in confirming the significant contribution of the DCP to oxygen requirements of photoreceptors in DMI, while highlighting the ability of AOSLO to detect subtle photoreceptor changes not always visible on SD-OCT.

## Introduction

Diabetic macular ischemia (DMI) is one of the major complications of diabetic retinopathy (DR) leading to severe loss of visual acuity [1]. Fluorescein angiography (FA) shows enlargement of the foveal avascular zone (FAZ) [2] and macular capillary closure in eyes with DMI [3]. While DMI may be associated with diabetic macular edema (DME) [4,5], in about 15% of cases, however, DMI becomes the predominant feature of the retinopathy leading to vision loss that cannot be explained by DME or neovascular complications [6,7]. In a recent study, Sim et al found that eyes with extensive DMI had significant thinning of the outer retina, suggesting that photoreceptor disruption may contribute to the associated vision loss [8].

By correcting the wavefront aberrations of the eye, adaptive optics scanning laser ophthalmoscopy (AOSLO) enables visualization of individual cone photoreceptors [9]. On AOSLO, photoreceptors appear as bright spots due to their waveguiding properties; light is directed by the inner segment into the outer segment, and then reflected back toward the pupil center [10]. Disruption of the waveguiding properties of photoreceptors on AOSLO has been shown to correspond to disruption of the photoreceptor layers on spectral domain-optical coherence tomography (SD-OCT) [11]. Previous studies using various AO techniques to assess the outer retina in patients with DR have shown decreased perifoveal photoreceptor density [12–14], a higher spread in neighbor distances between cones and a deviation from the normal cone packing arrangement [14]. To our knowledge, none of these studies have explored the correlation between photoreceptor changes on AO and the extent of overlying retinal capillary non-perfusion.

In a previous study, we found that retinal capillary non-perfusion on FA corresponded with outer retinal disruption on SD-OCT in eyes with DMI [15]. Then, we used OCT angiography (OCTA) to confirm that non-flow at the overlying deep retinal capillary plexus (DCP) correlated tightly to these zones of photoreceptor abnormalities on SD-OCT [16]. In the current study, we use AOSLO with OCTA to further test the hypothesis that non-flow at the level of the DCP is associated with photoreceptor abnormalities in patients with DR. We discuss these findings and their pathophysiologic significance in relationship to previous experimental studies of the effects of hypoxia on cone outer segments.

## Methods

Patients were prospectively recruited in the Department of Ophthalmology at Northwestern University in Chicago, Illinois between May 27, 2015 and June 10, 2016. This study was approved by the Institutional Review Board of Northwestern University and followed the tenets of the Declaration of Helsinki, and was performed in accordance with the Health Insurance Portability and Accountability Act regulations. Written informed consent was obtained from all participants.

### Study sample

Inclusion criteria for this study required a diagnosis of diabetic retinopathy, ranging from minimal nonproliferative (NPDR) to high risk and quiescent proliferative (PDR), based on the grading of color fundus photographs [17] analyzed by one experienced retinal specialist (A.A.F.), and the ability to obtain high-quality AOSLO and OCTA images. We included patients with both type 1 and type 2 diabetes mellitus (DM).

Exclusion criteria included eyes that have undergone surgical retinal repair, received intravitreal anti-vascular endothelial growth factor or laser treatment in the macula. We specifically excluded eyes with DME, which was evaluated using SD-OCT. We included eyes with minimal retinal hard exudates (four eyes), but these exudates were located outside the area of study. We excluded eyes with significant hemorrhages, astigmatism (> 3 diopters), or cataracts in order to avoid optical artifacts. We excluded eyes with any other known retinal diseases. After image acquisition, we excluded eyes where the OCTA images had movement or shadow artifacts, OCTA signal strength score lower than 50, and eyes with previously undetected macular edema. Finally, we excluded eyes with AOSLO images that were not gradable. Electronic medical records were reviewed to extract demographic and clinical information.

### Outer retinal evaluation

Outer retinal abnormalities were evaluated using the SD-OCT B-scans from the RTVue-XR Avanti OCTA system (Optovue Inc., Fremont, California). In one patient who did not have OCTA images, we evaluated SD-OCT from the Spectralis HRA+OCT imaging device (HRA; Heidelberg Engineering, Heidelberg, Germany). The imaging protocol for the Heidelberg SD-OCT included 31 horizontal B-scans per volume scan spanning 20° x 20° of the macula, each B-scan including an average of 30 frames. The tracking feature of the Heidelberg Spectralis HRA+OCT allowed each OCT B-scan to be registered to its exact location on the infrared reflectance (IR) image.

A trained retina specialist (A.A.F.) graded the outer retina on SD-OCT as “normal” or “abnormal”. Outer retinal abnormalities were defined as focal absence or reduced reflectivity of the inner segment / outer segment (IS/OS) junction or the outer segment / retinal pigment epithelium (OS/RPE) junction. We also carefully inspected the SD-OCT B-scans on both the Optovue device and the Spectralis device to ensure any findings of photoreceptor abnormalities or capillary non-flow were not due to shadowing artifacts related to overlying retinal pathology (i.e., hard exudates) or media opacity.

### Angiographic imaging

We obtained 3 x 3 mm^2^ OCTA images centered on the fovea using the RTVue-XR Avanti system (Optovue Inc., Fremont, California) with split-spectrum amplitude-decorrelation angiography (SSADA) software [18,19]. This instrument has an A-scan rate of 70,000 scans per second and uses a light source centered on 840 nm and a bandwidth of 45 nm. The SSADA algorithm detects flow by quantifying the decorrelation of the OCT reflectance between two consecutive B-scans at the same location on the retina. *En face* OCT angiograms were automatically segmented to define the superficial capillary plexus (SCP) and DCP. We then used the built-in AngioVue Analytics software (version 2016.1.0.26) to identify regions of capillary non-perfusion, defined as areas of “non-flow” larger than 100 microns in diameter. We also used AngioVue Analytics to quantify the “parafoveal” vessel density of the DCP. The “parafovea” was defined as an annulus centered on the fovea with inner and outer ring diameters of 1 mm and 3 mm, respectively. Vessel density was defined as the area occupied by vessels and microvasculature, and is reported as a percentage of the total area. To calculate vessel density, the AngioVue Analytics software extracts a binary image of the blood vessels from the grayscale OCTA image, and then calculates the percentage of pixels occupied by blood vessels in the defined region. In one eye, OCTA was not available at the time of examination and FA was used instead to define areas of macular non-perfusion. *En face* structural OCT images segmented to include the IS/OS and OS/RPE were also obtained from the OCTA to correlate with AOSLO.

### Adaptive optics imaging

AOSLO imaging was performed using the Apaeros retinal imaging system (Boston Micromachines Corporation, Boston, Massachusetts). Two superluminescent diodes (SLD) were used as light sources, centered at 780 and 830 nm with bandwidths of 20 and 15 nm, respectively. The power at the eye was approximately 200 microwatts. A tweeter mirror (Boston Micromachines Corporation, Boston, Massachusetts) with 140 actuators and 5.5 μm of stroke corrected for higher order aberrations, while a woofer mirror (AlpAO SAS, Montbonnot, France) with 37 actuators and 25 μm of stroke corrected lower order aberrations.

Adequate pupillary dilation was ensured prior to imaging, which began in the fovea and continued in a grid or line pattern steered by a wide field mirror. Focusing on the photoreceptors, the investigator acquired 2° x 2° images of the retina, as well as 1° x 1° images in areas of interest to provide high-resolution insets. We acquired 100 frames (individual images) per area of retina. Steps of 0.5° between each 2° x 2° patch of retina were taken to ensure adequate overlap for montaging purposes.

### Adaptive optics image processing and grading

Each block of frames corresponding to a 2° x 2° or 1°x 1° location was averaged using a custom MATLAB (Mathworks, Inc., Natick, Massachusetts) program [20]. This software utilizes algorithms to dewarp and align frames, and then compresses each block into an averaged, high-resolution image. The resulting 2° x 2° images were exported into i2k Retina Pro montaging software (DualAlign LLC, Clifton Park, NY, USA) for automated montaging.

**Fig 1:**
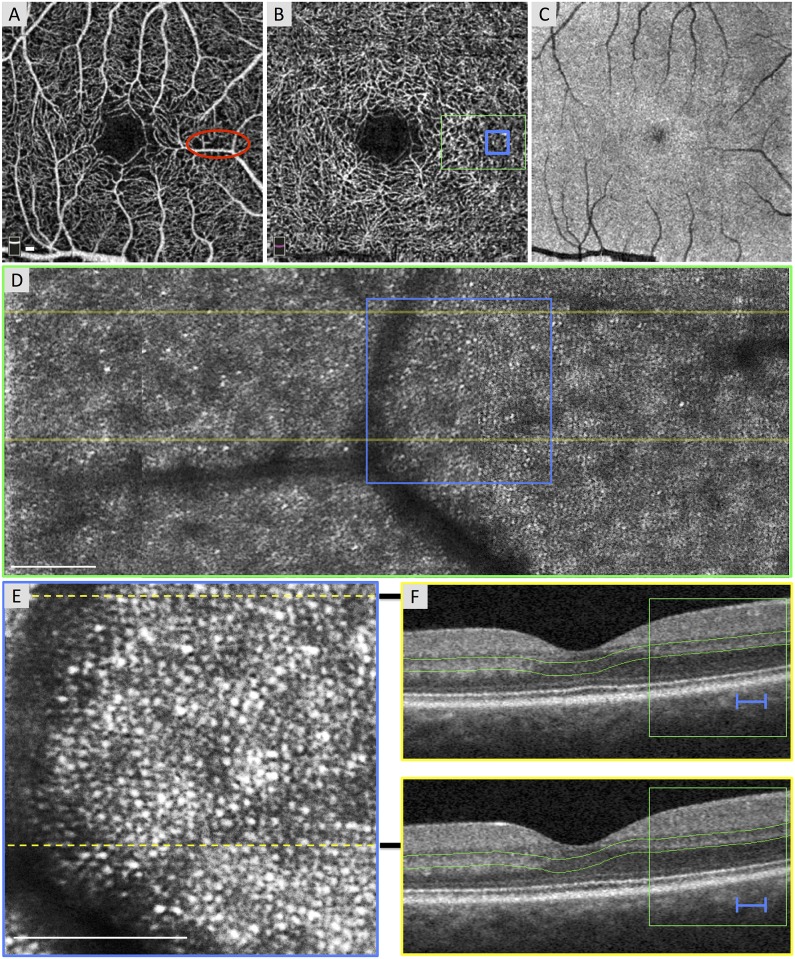
Normal Photoreceptors in an Area of Non-Flow of the Superficial Capillary Plexus (SCP). Case 1, right eye. (A) Optical coherence tomography angiography (OCTA) of the SCP shows a relatively normal contour of the foveal avascular zone (FAZ) with focal areas of capillary non-flow inferior and superior to the FAZ, including an area imaged by adaptive optics scanning laser ophthalmoscopy (AOSLO) (red circle). (B) OCTA of the deep capillary plexus (DCP) with location of AOSLO montage (green box) and enlarged inset (blue box). DCP shows a normal FAZ, robust capillaries throughout, and a vessel density of 63.46%. (C) *En face* structural OCT image segmented at the inner segment / outer segment (IS/OS) and the outer segment / retinal pigment epithelium (OS/RPE) junctions is unable to resolve the photoreceptor mosaic. (D) AOSLO montage stitched from 2° x 2° images with location of B-scans (yellow lines) and enlarged inset below (blue box). (E) Enlarged 1° x 1° AOSLO image from montage with heterogeneity packing index of 0.432. Dotted lines indicate location of B-scans. (F) Spectral domain (SD)-OCT from the OCTA device showing robust IS/OS and OS/RPE bands. Green box and blue lines show location of AOSLO montage and enlarged inset, respectively. Green lines indicate the segmentation boundaries for the DCP. White scale bars in A, D and E are 100 μm.

We examined the AOSLO images for outer segment signal to identify discontinuity in the photoreceptor mosaic. Photoreceptors were defined as normal or waveguiding on AOSLO if they were visible and hyper-reflective, appearing as a mosaic of bright, distinguishable dots (Fig 1). To exclude the potential confounding factor of improper image focus and other image acquisition confounders, we additionally required the presence of normal photoreceptors within the same frame for confirmation of focal photoreceptor abnormalities.

We assessed the cone packing arrangement using Voronoi diagrams, which we implemented with the *voronoi* MATLAB function. We created one Voronoi diagram per eye: one in the area of DCP non-flow for the eyes with DCP non-flow, and one area at a similar eccentricity for the eyes with DR without DCP non-flow. For Voronoi analysis, we used AOSLO images that were 200 by 200 pixels, obtained from images covering 1° x 1° of the retina taken at 2° to 3° from the foveal center (or about 578 to 867 μm from the foveal center in an eye with a focal length of 16.7 mm). Each Voronoi cell was coded by a different shade of gray corresponding to the number of neighboring cones. From each Voronoi diagram, we calculated the heterogeneity packing index (HPi), developed by Lombardo and colleagues, which represents the increase in 4- and 8-sided cones compared to 6-sided cones [14]. A lower HPi represents a greater deviation from the normal packing arrangements of cone photoreceptors. Before calculating the HPi, we manually checked each image to ensure the algorithm properly identified each cone photoreceptor.

### Image overlay

After image grading, all AOSLO images were uniformly enhanced for cone visualization by increasing the brightness, contrast and sharpness by 22%, 11% and 37%, respectively. Using retinal vascular landmarks as guides, AOSLO montages were then manually overlaid onto the OCTA images to allow correlation between DCP non-perfusion, and outer retinal abnormalities on SD-OCT and AOSLO. For the one patient without OCTA, we overlaid the FA and AOSLO images onto the IR image and then used Heidelberg Eye Explorer (version 1.7.0.0, Spectralis Viewing Module 5.4.6.0; Heidelberg Engineering, Heidelberg, Germany) to perform correlations between the FA, SD-OCT and AOSLO images.

### Statistics

Statistical analysis was performed using the SPSS software (version 17.0; SPSS, Inc., Chicago, IL). We ran independent samples t-tests for all numerical demographic information (Table 1). We also ran independent samples t-tests to assess any differences between eyes with and eyes without DCP non-flow for parafoveal DCP vessel density and for HPi. We used a Spearman rank test to determine the correlation coefficient between photoreceptor HPi and DCP parafoveal vessel density.

**Table 1:** Characteristics of Study Participants with Diabetic Retinopathy.

Case #	*Sex/Age*, *y*	*DM Type*	*Duration of DM*, *y*	*HbA1c*	*Study Eye*	*BCVA*	*DR Stage*	*Laser Treatment*	*Diabetes Medication*	*DCP Density (%)*	*HPi*
**Normal DCP**											
1	M/48	1	7	12.0	Right	20/20	NPDR	None	Insulin, glucagon	63.46	0.432
2	M/37	2	6	8.1	Right	20/15	NPDR	None	Insulin	58.02	0.429
3	F/33	1	26	9.0	Right	20/20	PDR	None	Insulin	58.94	0.433
**DCP Non-Flow**											
4	F/33	1	25	8.0	Left	20/20	PDR	PRP	Insulin, Metformin	55.81	0.366
5	F/31	1	17	5.8	Right	20/20	PDR	None	Insulin	51.44	0.429
6	F/68	2	28	10.4	Right	20/50	PDR	PRP	Insulin	46.39	0.321
7	F/53	2	8	6.9	Right	20/25	NPDR	None	Insulin	-	0.328
8	M/43	2	6	7.4	Left	20/25	PDR	PRP	Insulin	49.52	0.314
9	M/47	2	12	8.2	Left	20/25	PDR	None	Insulin	42.96	0.397
10	F/33	2	5	7.2	Right	20/20	NPDR	None	Insulin	57.69	0.360
11	M/47	2	2	7.8	Right	20/30	PDR	PRP	Metformin	47.69	0.352
**Normal DCP (1–3)**											
Mean (SD)	39.3 (7.8)		13.0 (11.3)	9.7 (2.0)	-	-	-	-	-	60.14 (2.91)	0.431 (0.002)
**DCP Non-Flow (4–11)**											
Mean (SD)	44.4 (12.5)		12.9 (9.6)	7.7 (1.3)	-	-	-	-	-	50.21 (5.21)	0.358 (0.013)
*P*-value	*0*.*54*	*-*	*0*.*99*	*0*.*084*	*-*	*-*	*-*	*-*	*-*	*0*.*016*	*0*.*013*

Deep capillary plexus parafoveal vessel density on optical coherence tomography angiography and the cone photoreceptor heterogeneity packing index on adaptive optics scanning laser ophthalmoscopy were significantly reduced in patients with diabetic macular ischemia.

Abbreviations: BCVA = Best corrected visual acuity, DCP = Deep capillary plexus, DM = Diabetes mellitus, DR = Diabetic retinopathy, HbA1c = Glycated hemoglobin, percent of total hemoglobin, HLD = Hyperlipidemia, HPi = Heterogeneity Packing index, HTN = Hypertension, KD = Kidney disease, NPDR = Non-proliferative diabetic retinopathy, OU = Oculus uterque (both eyes), OD = Oculus dexter (right eye), OS = Oculus sinister (left eye), PDR = Proliferative diabetic retinopathy, PRP = Panretinal photocoagulation, SD = Standard deviation, y = Years, *P*-value = Independent samples t-test between Normal DCP and DCP non-flow groups for each parameter.

## Results

A total of 11 eyes of 11 patients with DR (median age, 43 years; average age, 43.3 years; range 31–68 years) were included in the analysis. The median duration of DM was eight years (average, 12.9 years; range, 2–28 years). Eight eyes showed evidence of DMI at the level of the DCP (eyes with DCP non-flow). Three eyes with DR showed normal macular perfusion of the DCP (eyes with DR without DCP non-flow). Table 1 summarizes the ocular findings and demographic characteristics by case number. The demographics of the eyes with DCP non-flow compared to eyes with DR without DCP non-flow were matched (Table 1).

### Vessel density and voronoi analysis

**Fig 2:**
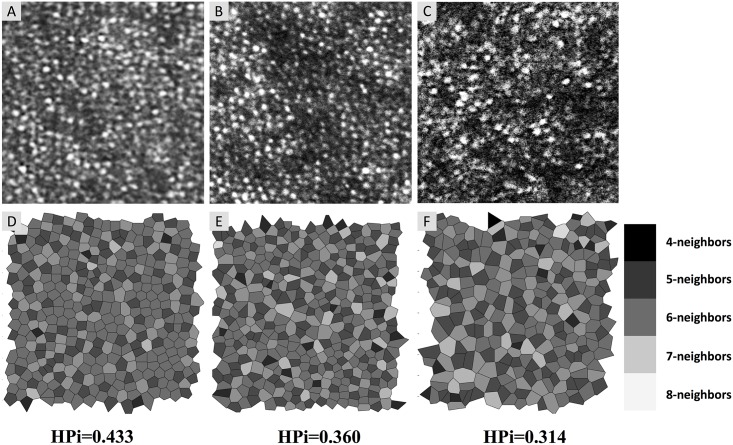
Adaptive Optics Scanning Laser Ophthalmoscopy (AOSLO) Images and Corresponding Voronoi Diagrams with Heterogeneity Packing Index (HPi). (A-C) AOSLO images of cone mosaic (200 by 200 pixels taken from 1° x 1° images). (D-F) Voronoi tessellation corresponding to the image above with shading of cells indicating the number of neighboring photoreceptor cells, from dark (four neighbors) to light (eight neighbors). A lower HPi represents a larger deviation from the normal packing arrangement of cones. (A and D) Cones in an eye with DR without deep capillary plexus (DCP) non-flow (Case 3, HPi = 0.433). (B and E) Cones in an area of DCP non-flow (Case 10, HPi = 0.360). (C and F) Cones in an area of DCP non-flow (Case 8, HPi = 0.314).

**Fig 3:**
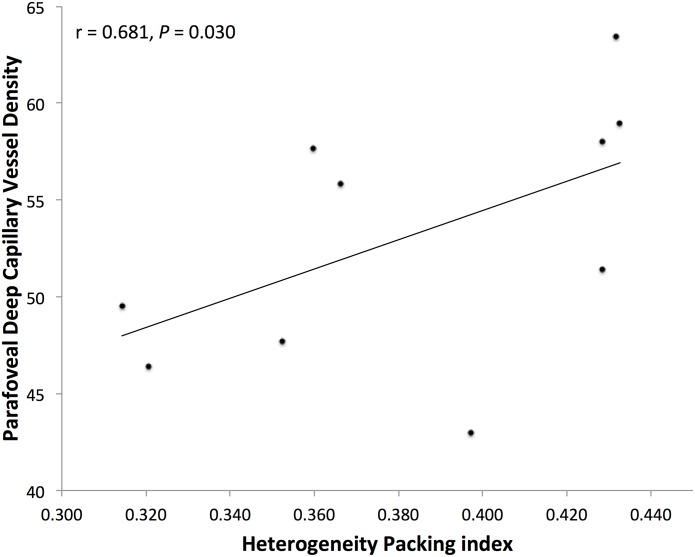
Heterogeneity Packing Index (HPi) of Cones was Significantly Correlated with Deep Capillary Plexus (DCP) Vessel Density. The Spearman rank test showed a significant correlation between HPi from adaptive optics scanning laser ophthalmoscopy imaging and parafoveal DCP vessel density from optical coherence tomography angiography (r = 0.681, *P* = 0.030) for the ten eyes with both types of imaging performed.

We found a significant reduction in parafoveal DCP vessel density in eyes with DCP non-flow compared to eyes with DR without DCP non-flow (50.21% ± 5.21% for DCP non-flow, and 60.14% ± 2.91% for DR without DCP non-flow; *P* = 0.016; Table 1). For the Voronoi analysis of cones, we found a significant reduction in HPi in eyes with DCP non-flow compared to eyes with DR without DCP non-flow (0.358 ± 0.013 for DCP non-flow, and 0.431 ± 0.002 for DR without DCP non-flow; *P* = 0.013; Table 1). Fig 2 shows cones with Voronoi diagrams and HPi for an eye with DR without DCP non-flow, and two eyes with DCP non-flow. A significant correlation was found between photoreceptor HPi and parafoveal DCP vessel density for the ten eyes that had both OCTA and AOSLO (r = 0.681, *P* = 0.030; Fig 3).

### Local analysis

Three eyes (Cases 1–3) with DR without DCP non-flow had a normal FAZ contour and robust DCP capillary perfusion, and showed consistently higher HPi than eyes with DCP non-flow (Fig 1 and Table 1). All three eyes showed normal IS/OS and OS/RPE junctions on SD-OCT. Two of the eyes showed focal areas of capillary non-flow at the level of the SCP with normal underlying DCP and photoreceptors (Fig 1).

**Fig 4:**
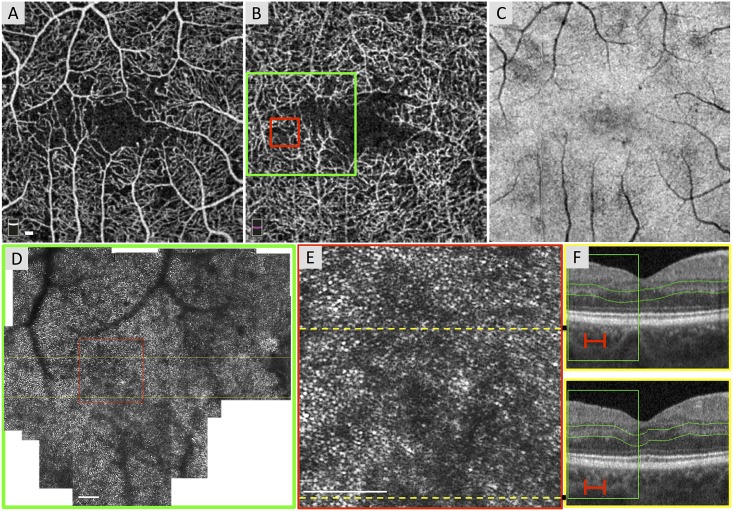
Reduced Photoreceptor Heterogeneity Packing Index (HPi) in an Area of Capillary Non-Flow of the Deep Capillary Plexus (DCP). Case 4, right eye. (A) Optical coherence tomography angiography (OCTA) of the superficial capillary plexus (SCP) shows a relatively normal foveal avascular zone (FAZ), along with distinct foci of capillary non-flow throughout angiogram. (B) OCTA of the DCP with location of adaptive optics scanning laser ophthalmoscopy (AOSLO) montage (green box). DCP reveals an enlarged and irregular FAZ contour and has a vessel density of 55.81%. The red box highlights an area of capillary non-flow and the location of the enlarged AOSLO inset. (C) *En face* structural OCT image segmented at the inner segment / outer segment (IS/OS) and the outer segment / retinal pigment epithelium (OS/RPE) junctions cannot resolve photoreceptor integrity. (D) AOSLO montage stitched from 2° x 2° images with location of B-scans (yellow lines) and enlarged inset (red box). (E) Enlarged 1° x 1° AOSLO image from montage (HPi = 0.366). Dotted lines indicate location of B-scans. (F) Spectral domain (SD)-OCT from the OCTA device. Green box and red lines show location of AOSLO montage and enlarged inset, respectively. The IS/OS and OS/RPE bands appear normal. Green lines indicate the segmentation boundaries for the DCP. White scale bars in A, D and E are 100 μm.

**Fig 5:**
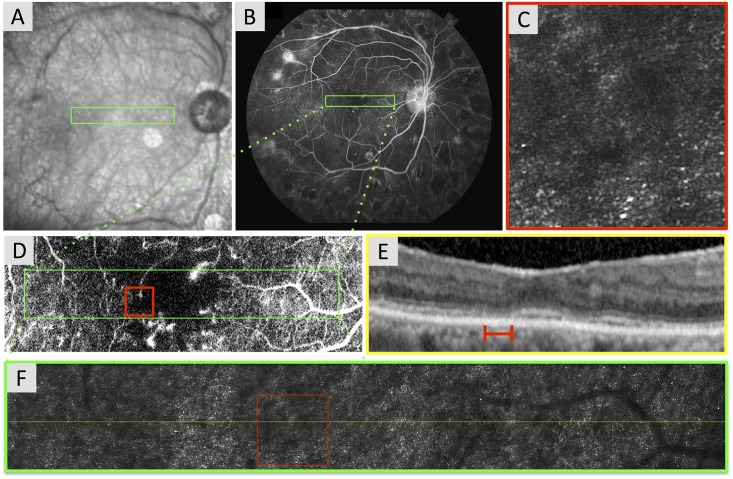
Reduced Photoreceptor Heterogeneity Packing Index (HPi) in an Eye with Capillary Non-Perfusion Contiguous with the Foveal Avascular Zone (FAZ). Case 7, right eye. (A) Infrared (IR) image with location of adaptive optics scanning laser ophthalmoscopy (AOSLO) montage (green box). (B) Fluorescein angiography (FA) with location of AOSLO montage (green box). (C) Enlarged 1° x 1° AOSLO image from montage (HPi = 0.328). (D) Enlarged FA from B reveals an enlarged and irregular contour of the FAZ with surrounding contiguous areas of capillary non-perfusion. Green box shows location of AOSLO montage in F. Red box shows the location of enlarged AOSLO image (C) in the area of the enlarged FAZ. (E) Spectral-domain optical coherence tomography (SD-OCT) registered to the IR image showing the retinal area covered by the AOSLO montage. B-scan shows focal points where inner segment / outer segment junction is interrupted with decreased intensity of the outer segment / retinal pigment epithelium junction. Red line shows location of enlarged AOSLO image in C. (F) AOSLO montage stitched from 2° x 2° images with location of OCT B-scan (yellow line) and enlarged AOSLO inset (red box).

In eight eyes with DCP non-flow (Cases 4–11), areas of DCP ischemia corresponded to zones of reduced photoreceptor HPi on AOSLO (Figs 4 and 5, S1 and S2 Figs). Seven of the eyes with DCP non-flow had OCTA images, which showed ischemia localized to the level of the DCP (with or without SCP involvement). This included irregular and enlarged contour of the FAZ, and/or areas of DCP non-flow in the parafovea. For the eye without OCTA, an irregular and enlarged FAZ contour was seen on FA, which indicated overlapping SCP and DCP ischemia, by definition (Fig 5).

In six of eight eyes with DCP non-flow, zones of photoreceptor abnormalities (reduced HPi) corresponded tightly to areas of IS/OS and OS/RPE abnormalities on SD-OCT (Fig 5 and S2 Fig). In contrast, two eyes with photoreceptor abnormalities on AOSLO had normal appearing photoreceptor layers on SD-OCT (Fig 4 and S1 Fig). Furthermore, zones of photoreceptor abnormalities on AOSLO in four eyes correlated tightly to hypo-reflectivity on *en face* SD-OCT images segmented at the IS/OS and OS/RPE (S2 Fig). In six eyes with DCP ischemia, some capillaries seen in the DCP were verified, by comparing the SCP to DCP, to be artifact projections from the SCP (Fig 4, S1 and S2 Figs). This type of projection artifact may lead to an underestimation of the extent of DCP non-perfusion when using OCTA [21].

## Discussion

In the current study, we confirm the hypothesis that DCP ischemia is associated with abnormalities of cone photoreceptor layer in DR as revealed on AOSLO. We found that eyes with DCP non-flow had abnormal cone packing arrangements (lower HPi) compared to eyes with DR without DCP ischemia, and that DCP vessel density correlated significantly with HPi. We also found that areas of photoreceptor abnormalities on AOSLO corresponded to abnormalities of the photoreceptor lines on SD-OCT in some eyes, while these photoreceptor changes on AOSLO were not detectable on OCT in others.

In recent studies, we have used SD-OCT scans to show outer retinal disruption that co-localized to non-perfusion on FA [15] and specifically to the DCP on OCTA in eyes with DMI [16]. While OCT provides excellent axial resolution, its lateral resolution is limited to > 20 microns due to monochromatic aberrations of the eye [22]. In comparison, by correcting these aberrations, AOSLO allows *en face* assessment of individual photoreceptor cells [23,24]. In the current study, we confirm that the abnormalities of photoreceptor lines we observed on OCT correspond to zones of abnormal cones on AOSLO. Furthermore, we have demonstrated the ability of AOSLO to detect photoreceptor abnormalities that are not visualized on SD-OCT (Fig 4 and S1 Fig).

Fluctuations in cone reflectivity on AOSLO, which can influence HPi calculations, may be caused by molecular changes within the cell during various states of photo-transduction or changes in outer segment length [25]. Reduced cone reflectivity may also indicate morphological alterations that interfere with their waveguiding abilities [26]. Some studies have shown that increased cone spacing and reduced outer segment reflectivity on AOSLO correspond to areas of visual defects in microperimetry [27–29] as well as reduced amplitude and response densities on multifocal electroretinography [30,31]. However, the correlation between cone reflectivity and cone function is unclear, as even cones with low reflectivity on AOSLO can have normal sensitivity to light. [32]

To our knowledge, this is the first study that utilizes AO to study photoreceptors in areas of DCP non-perfusion. In a previous flood-illumination AO study, Lombardo et al found an average of 10% decrease in parafoveal cone density in eyes with type 1 DM and a mean duration of diabetes of 13 years [12]. Lombardo also identified pathological disruptions of the parafoveal cone mosaic in patients with DM by using cone density, linear dispersion index, and HPi parameters [14]. In this latter study, Lombardo found significantly decreased HPi in eyes of patients with DM, both with and without DR, compared to healthy controls. Lammer et al studied a number of different AOSLO parameters that measure the regularity cone photoreceptor packing arrangements in healthy patients and patients with diabetes (No DR, NPDR, and PDR). The authors found that a decrease in some of these parameters was associated with the presence of DM, with increased DR severity, and with the presence of edema [33]. While these previous studies provide important insights into the status of photoreceptors in eyes with DR, they did not study the status of the retinal circulation in relationship to the photoreceptor compromise. In the current study, we found a significant decrease in HPi in eyes with DCP non-flow compared to eyes with DR and normal DCP perfusion. Lower HPi indicates a deviation from the normal hexagonal packing arrangements of cones in the human retina [14,34]. The correlation of these areas to DCP non-flow in our study may suggest that outer retinal hypoxia contributed to the abnormal packing arrangement (likely associated with cone loss, as discussed in [14]), seen in these eyes [35–40].

Photoreceptors in rats have been shown to be specifically vulnerable to hypoxemia [35]. Prior to apoptosis, hypoxic photoreceptors deconstruct their outer segments to reduce their energy consumption [37,38]. These structural changes and apoptosis are greatly attenuated with induced hyperoxia in the setting of retinal detachment (RD), suggesting that oxygen deprivation plays a key role in this process [38–40]. Histological evidence from RD studies in the cat show that outer segments appear distorted, with progressive deterioration as the RD persists [41]. We believe that our current work may provide some insight into the pathological response of the photoreceptors to a different type of hypoxic condition in the living human retina, related to DCP ischemia.

The retina has a dual vascular supply from the retinal and choroidal circulations. The retinal vasculature in the macula is a complex system consisting of three capillary plexuses that do not extend deeper than the outer border of the inner nuclear layer, placing the photoreceptors in a “watershed zone”, where both the choroidal and retinal circulations may provide oxygen support [21,42]. While the outer retina is primarily dependent on diffusion from choroidal circulation for its oxygen demand [43], experimental studies have shown that photoreceptors rely on the retinal circulation for 10–15% of their oxygen needs [44]. Furthermore, blunted retinal autoregulation [45] and decreased retinal oxygen tension in the setting of DR may leave photoreceptors particularly susceptible to ischemic insults affecting the retinal circulation [46].

Limitations of our study include a small number of eyes. This was related to the difficulties acquiring high-resolution imaging in advanced DR, due to media opacities and vitreous hemorrhage. Furthermore, optical distortions (i.e., wavefront aberration, scattering, dispersion) have been shown to be significantly higher in eyes with DR, resulting in suboptimal AO performance [47]. In fact, both AOSLO and OCTA are highly susceptible to artifacts, but we took strict measures in our study design to reduce the potential for artifacts. In the current work, we excluded retinal regions with significant overlying optical obstructions and eyes with significant media opacity, as well as eyes with edema. This significantly decreased the number of patients eligible for the study, but strengthened our study by reducing potential confounding variables. Yet, since we used conventional imaging modalities to exclude eyes with these findings, it is possible that subtle pathological changes (i.e. subtle edema, microaneurysms, small hemorrhages, and lipid exudates), which are below the limits of resolution of standard imaging techniques, could have been present and may have affected the brightness of cones and cone visibility in AOSLO images.

This study was also limited by the extent of quantitative analyses. For example, we did not perform cone reflectance or SD-OCT outer retinal abnormality quantifications, but as this was a pilot study, these parameters may be appropriate for future studies. We also were unable to quantify eccentricity in millimeters from the fovea for each area of study since we did not obtain axial length measurements. Yet, we attempted to compare regions of similar eccentricity and only included individual AOSLO images with visible, resolved photoreceptors somewhere within the image. Without axial length, we were also unable to perform cone density measurements. Finally, OCTA is a relatively new technique, which needs larger cohort studies to understand the reliability of the method. Yet, in a study of 135 eyes of healthy adults, the Optovue OCTA device achieved good reproducibility, with an intraclass correlation coefficient of 0.81 for intra-session inter-observer reproducibility and 0.74 for inter-session intra-observer reproducibility for the parafoveal DCP vessel density measurement [48].

In conclusion, using AOSLO, this study confirms that abnormal cone packing arrangements are found in eyes with non-flow at the level of the DCP. This observation improves our understanding of the complex pathogenesis of poor visual prognosis associated with DMI. We believe that the use of AOSLO and OCTA, in combination, provides a novel approach to studying the pathophysiology of DMI at the microvascular and cellular level. Further studies utilizing OCTA and split-detector AOSLO, with the addition of quantitative data, including microperimetry, will be important to evaluate the structural and functional consequences of these interactions. The recent development of visible light-OCT for *in vivo* human retinal metabolic imaging will also allow us to gain a deeper insight into these metabolic derangements [49].

## Supporting Information

S1 Fig Deep Capillary Plexus (DCP) Non-Flow Associated with Low Photoreceptor Heterogeneity Packing Index (HPi) on Adaptive Optics. Case 5, left eye. (A) Optical coherence tomography angiography (OCTA) of the superficial capillary plexus (SCP) shows an irregular contour of the foveal avascular zone (FAZ) with contiguous capillary non-flow temporally. (B) OCTA of the DCP with location of adaptive optics scanning laser ophthalmoscopy (AOSLO) montage (green outline) and enlarged inset (red box). DCP angiogram shows capillary non-flow areas, especially temporal to the fovea (red box), and has a vessel density of 51.44%. (C) *En face* structural OCT image segmented at the inner segment / outer segment (IS/OS) and the outer segment / retinal pigment epithelium (OS/RPE) junctions cannot resolve the photoreceptor integrity. (D) AOSLO montage stitched from 2° x 2° images with location of B-scans (yellow lines) and enlarged inset (red box). (E) Enlarged 1° x 1° AOSLO image from montage (HPi = 0.429). Dotted lines indicate location of B-scans. (F) Spectral domain (SD)-OCT from the OCTA device. Red lines show location of AOSLO enlarged inset. The IS/OS and OS/RPE bands appear normal. Green lines indicate the segmentation boundaries for the DCP. White scale bars in A, D and E are 100 μm.
(TIF)

S2 Fig Reduced Photoreceptor Heterogeneity Packing Index (HPi) Corresponds to Defect on *En Face* Structural Optical Coherence Tomography (OCT). Case 6, right eye. (A) OCT angiography (OCTA) of the superficial capillary plexus (SCP) shows an enlarged and irregular contour of the foveal avascular zone (FAZ) and focal capillary non-flow surrounding the FAZ. (B) OCTA of the deep capillary plexus (DCP) with location of adaptive optics scanning laser ophthalmoscopy (AOSLO) montage (green outline) and enlarged inset (red box). DCP angiogram shows capillary non-flow in same areas as SCP non-flow, although the majority of the capillaries in the DCP slab that are within the green AOSLO outline appear to be projection artifacts from the SCP. DCP vessel density was 46.39%. (C) *En face* structural OCT image segmented at the inner segment / outer segment (IS/OS) and the outer segment / retinal pigment epithelium (OS/RPE) junctions cannot resolve individual photoreceptor integrity, but dark areas on *en face* OCT (red box) correspond to a zone of reduced photoreceptor HPi in E. (D) AOSLO montage stitched from 2° x 2° images with location of B-scan (yellow line) and enlarged inset (red box). (E) Enlarged 1° x 1° AOSLO image from montage (red box). Within the area of DCP non-flow, this location has a photoreceptor HPi of 0.321. Dotted line shows location of B-scan. (F) Spectral domain (SD)-OCT from the OCTA device. Green box and red line show location of AOSLO montage and enlarged inset, respectively. The IS/OS and OS/RPE bands appear abnormal and hypo-reflective. Green lines indicate the segmentation boundaries for the DCP. White scale bars in A, D and E are 100 μm.
(TIF)
