# Supplementary figures and images for: Adaptive Optics Reveals Photoreceptor Abnormalities in Diabetic Macular Ischemia
Source: PLoS One. 2017 Jan 9;12(1):e0169926. doi: 10.1371/journal.pone.0169926 (PMC5222506; doi:10.1371/journal.pone.0169926)

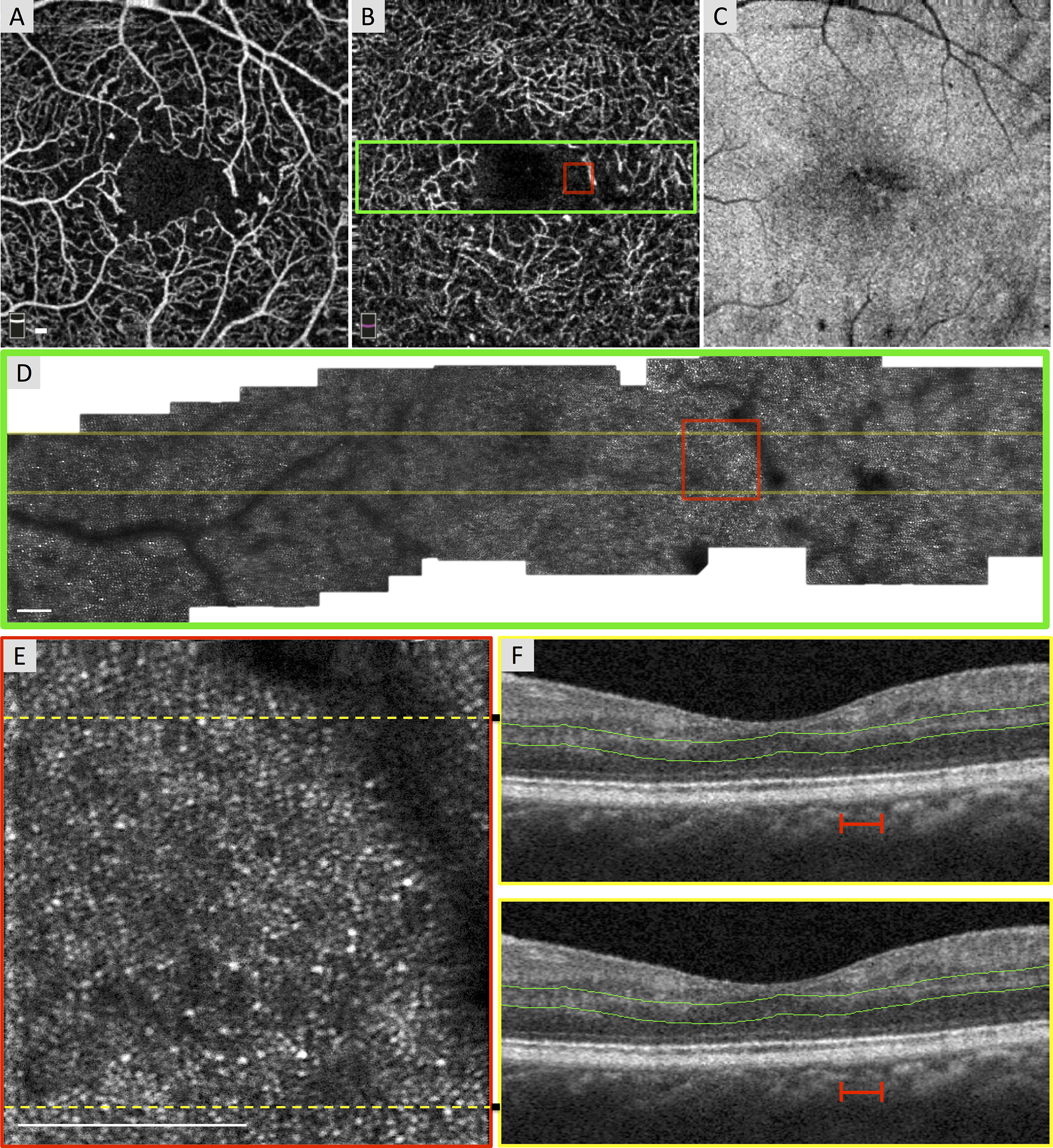

Supplement: S1 Fig — Case 5, left eye. (A) Optical coherence tomography angiography (OCTA) of the superficial capillary plexus (SCP) shows an irregular contour of the foveal avascular zone (FAZ) with contiguous capillary non-flow temporally. (B) OCTA of the DCP with location of adaptive optics scanning laser ophthalmoscopy (AOSLO) montage (green outline) and enlarged inset (red box). DCP angiogram shows capillary non-flow areas, especially temporal to the fovea (red box), and has a vessel density of 51.44%. (C) En face structural OCT image segmented at the inner segment / outer segment (IS/OS) and the outer segment / retinal pigment epithelium (OS/RPE) junctions cannot resolve the photoreceptor integrity. (D) AOSLO montage stitched from 2° x 2° images with location of B-scans (yellow lines) and enlarged inset (red box). (E) Enlarged 1° x 1° AOSLO image from montage (HPi = 0.429). Dotted lines indicate location of B-scans. (F) Spectral domain (SD)-OCT from the OCTA device. Red lines show location of AOSLO enlarged inset. The IS/OS and OS/RPE bands appear normal. Green lines indicate the segmentation boundaries for the DCP. White scale bars in A, D and E are 100 μm. (TIF) [file pone.0169926.s001.tif]

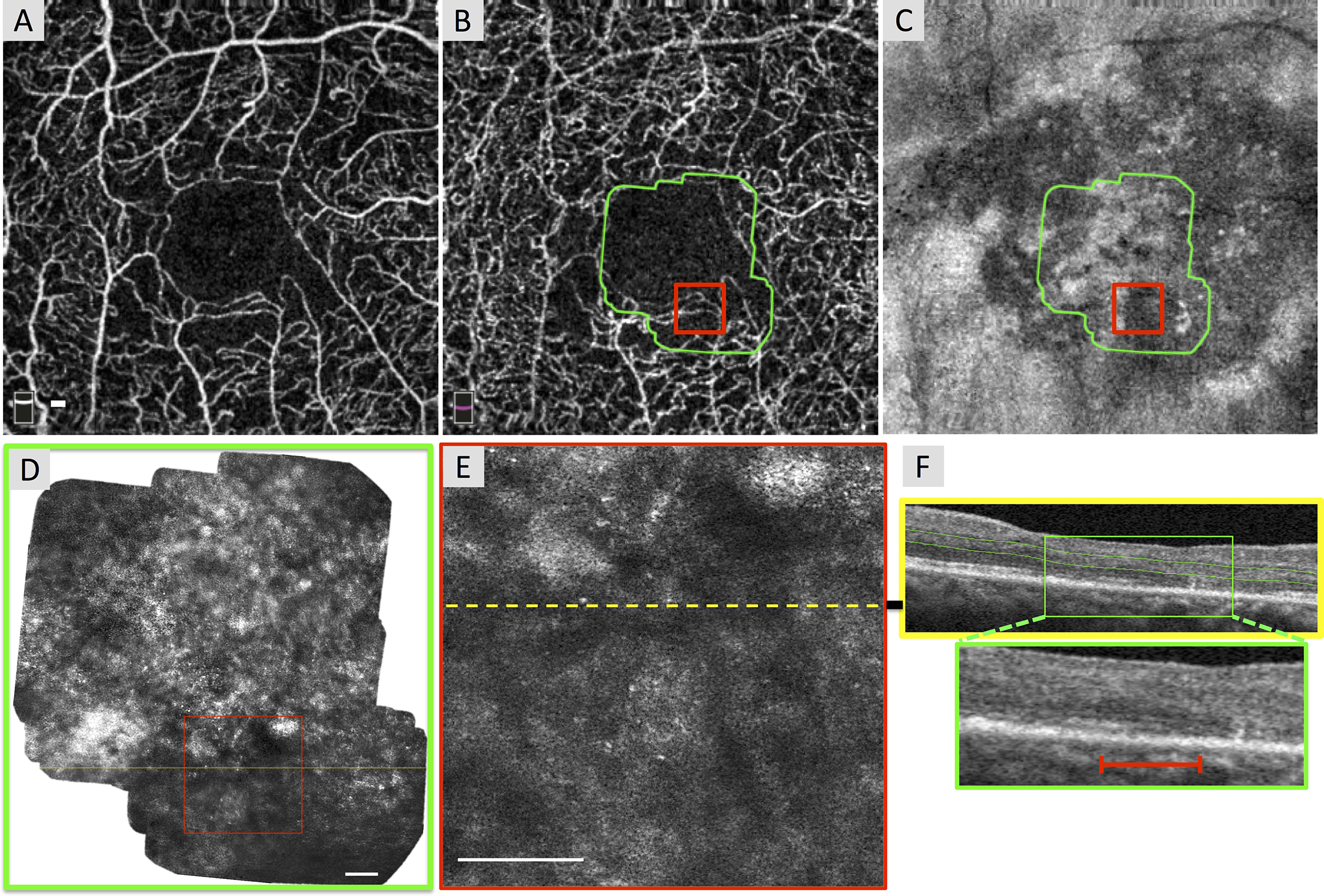

Supplement: S2 Fig — Case 6, right eye. (A) OCT angiography (OCTA) of the superficial capillary plexus (SCP) shows an enlarged and irregular contour of the foveal avascular zone (FAZ) and focal capillary non-flow surrounding the FAZ. (B) OCTA of the deep capillary plexus (DCP) with location of adaptive optics scanning laser ophthalmoscopy (AOSLO) montage (green outline) and enlarged inset (red box). DCP angiogram shows capillary non-flow in same areas as SCP non-flow, although the majority of the capillaries in the DCP slab that are within the green AOSLO outline appear to be projection artifacts from the SCP. DCP vessel density was 46.39%. (C) En face structural OCT image segmented at the inner segment / outer segment (IS/OS) and the outer segment / retinal pigment epithelium (OS/RPE) junctions cannot resolve individual photoreceptor integrity, but dark areas on en face OCT (red box) correspond to a zone of reduced photoreceptor HPi in E. (D) AOSLO montage stitched from 2° x 2° images with location of B-scan (yellow line) and enlarged inset (red box). (E) Enlarged 1° x 1° AOSLO image from montage (red box). Within the area of DCP non-flow, this location has a photoreceptor HPi of 0.321. Dotted line shows location of B-scan. (F) Spectral domain (SD)-OCT from the OCTA device. Green box and red line show location of AOSLO montage and enlarged inset, respectively. The IS/OS and OS/RPE bands appear abnormal and hypo-reflective. Green lines indicate the segmentation boundaries for the DCP. White scale bars in A, D and E are 100 μm. (TIF) [file pone.0169926.s002.tif]
